# Rarely Occurring Unusual Complications Following the Administration of the Inferior Alveolar Nerve Block: A Systematic Review of Case Reports

**DOI:** 10.7759/cureus.84966

**Published:** 2025-05-28

**Authors:** Vijaylaxmi Shettar, Alka Kale, Rajendra V Mali

**Affiliations:** 1 Oral and Maxillofacial Surgery, Karnataka Lingayat Education Vishwanath Katti (KLE VK) Institute of Dental Sciences, KLE Academy of Higher Education and Research (KAHER), Belagavi, IND; 2 Oral and Maxillofacial Pathology, Karnataka Lingayat Education Vishwanath Katti (KLE VK) Institute of Dental Sciences, KLE Academy of Higher Education and Research (KAHER), Belagavi, IND; 3 Radiology, Jawaharlal Nehru Medical College, Karnataka Lingayat Education (KLE) Academy of Higher Education and Research (KAHER), Belagavi, IND

**Keywords:** blanching, glossopharyngeal nerve palsy, inferior alveolar nerve block, laryngeal complications, neurological symptoms, rare complications, unusual complications

## Abstract

Unusual complications have been associated with the inferior alveolar nerve (IAN) block. In this study, we aimed to review and analyze the cases of rarely occurring unusual complications following the administration of the IAN block.

A systematic search of PubMed, Google Scholar, and Science Direct was conducted for articles on rarely occurring unusual complications following the administration of the inferior alveolar nerve block until September 2024, and articles documented in English were included. Based on inclusion and exclusion criteria, a total of 29 articles were selected, of which six articles could not be retrieved. A total of 23 articles were analyzed.

Various rare and unusual complications have been reported, which include Horner's syndrome, blanching of the face, self-inflicted injury, hoarseness, convulsions, headache, intracranial air embolism, glossopharyngeal nerve palsy, bleeding in the external auditory meatus, and herpes zoster infection. Treatment ranged from monitoring in mild cases to hospital admission in severe cases.

Awareness of these complications can help in better patient management. Precautions can be taken by dentists while administering the IAN block to avoid complications. These include the use of proper technique and management of patient anxiety. Rare, unusual complications must be considered when administering the IAN block.

## Introduction and background

The inferior alveolar nerve (IAN) block is the most commonly performed block in dentistry to anesthetize the lower teeth to perform various dental procedures. When administering the IAN block, the local anesthesia is deposited just before the nerve enters the mandibular foramen [1]. Anatomical variation and improper technique are some of the common causes for IAN block failure [2]. The position of the mandibular foramen can vary among individuals. This can result in the failure of the IAN block occasionally [3]. Various methods were tried to increase the anesthetic efficacy of the IAN block, e.g., patient position and needle bevel orientation. The pulpal anesthesia for premolars improved with the supine position. Anesthesia success remained unchanged with supine or upright position for molars and anterior teeth, with needle orientation toward or away from the mandibular ramus [4,5].

In 15.3% of cases of IAN block, the aspiration was positive [6]. Accidental intravascular injection of local anesthesia with vasoconstrictor can lead to complications [6].

Transient facial nerve palsy, hematoma, trismus, and ocular complications are some of the most common complications noted with IAN block [2,7-10].

The incidence of facial nerve palsy following the administration of IAN block is around 0.3% [11]. Difficulty in facial expression, inability to close the eyes, and drooping of the corner of the mouth on the affected side are noted in facial nerve palsy. Immediate or delayed type of facial nerve palsy has been reported. Immediate type is seen soon after the administration of local anesthesia. When the IAN block is administered posteriorly, the spread of the local anesthesia into the parotid gland, which incorporates the facial nerve, can lead to facial nerve palsy. This is transient in nature, and the condition reverses in a few hours [12]. Different theories have been proposed related to delayed-type facial nerve palsy. Delayed-type facial nerve palsy can result from a sympathetic vascular reflex due to ischemic paralysis at the entry of the stylomastoid foramen region. Other theories include stretching of the facial nerve due to prolonged mouth opening and reactivation of latent viral infections, such as herpes simplex virus or varicella zoster virus, due to trauma. The alcohols that form from the breakdown of local anesthesia may result in prolonged nerve damage. Pressure due to the backward flow of local anesthesia solution during intra-arterial injection can cause the solution to flow in different anatomical pathways; this could be another reason for delayed-type facial nerve palsy. The treatment of late facial nerve palsy involves steroids, eye protection by eye patch, and eye lubricant [13].

Damage to the blood vessel and subsequent bleeding in the surrounding tissues leads to a hematoma. Inferior alveolar artery damage during IAN block can result in a hematoma. If a hematoma occurs, the IAN block must be stopped immediately. Cold application, antibiotics, and analgesics must be administered, and the patient must be kept on follow-up for further management [7,14].

Repeated intramuscular injection, intramuscular hematoma due to inferior alveolar artery or vein injury, and needle track infection can lead to trismus on the administration of IAN block. Trismus resolves over weeks, and treatment involves a soft diet, muscle relaxants, etc. [15].

Needle breakage, another complication, was most commonly associated with the IAN block. Following the proper technique, advising patients to avoid sudden movements during the procedure, and avoiding needle bending or multiple injections can help prevent needle breakage during the administration of the IAN block. Early removal of a broken needle can help avoid complications. Some clinicians also suggested that a broken needle can be treated as a small, non-infected root tip, which can be left behind, and scar tissue will form around it; this prevents further complications. When needle breakage occurs, patients must be informed, and the event must be documented. Patients can be referred to higher centers. Proper investigations must be carried out to locate the needle, and treatment can be done accordingly [16].

Ocular complications, although rare, can occur while administering an IAN block. Ocular complications such as diplopia, amaurosis, and blurred vision have been reported. Most of these complications usually manifest immediately after the administration of local anesthesia. The majority of these are self-limiting, requiring only monitoring; in a few cases, additional treatment is required [8,9].

Unusual complications such as blanching, neurological complications, laryngeal complications, glossopharyngeal nerve palsy, and complications related to the external auditory meatus, although rare, have been reported in the literature following the administration of IAN block. The occurrence of an unexpected event during the administration of IAN block can make the dentist and patient anxious. Knowledge of the same can help dentists initiate the necessary measures to prevent a further sequence of serious events. Gathering all information related to unusual complications can lead to earlier diagnosis and management. Any pattern noted in the occurrence of complications in relation to patients with a particular medical history or any particular local anesthetic used could help in drawing inferences and conducting further studies.

In this study, we aimed to review and analyze cases of rare and unusual complications. Due to the rarity of the occurrence of unusual complications following the administration of IAN block, prospective studies are not feasible; hence, they are reported as case reports or case series.

## Review

Methods

Information Sources

A systematic search of PubMed, Google Scholar, and Science Direct was conducted for articles on rare and unusual complications following the administration of the IAN block. The last search was performed until September 2024.

Search Strategy

Search strategy was developed using keywords and MeSH terms related to "inferior alveolar nerve block", "complications", "unusual complications", "rare complications", "blanching", "neurological symptoms", and "glossopharyngeal nerve palsy", searched through the above databases.

PubMed Search Strategy

PubMed search strategy included ((inferior alveolar nerve block) AND (complication)) OR (unusual complication), (("mandibular nerve"[MeSH Terms] OR ("mandibular"[All Fields] AND "nerve"[All Fields])) OR "mandibular nerve"[All Fields] OR ("inferior"[All Fields] AND "alveolar"[All Fields] AND "nerve"[All Fields]) OR "inferior alveolar nerve"[All Fields]) AND ("block"[All Fields] OR "blocked"[All Fields] OR "blocking"[All Fields] OR "blockings"[All Fields] OR "blocks"[All Fields]) AND ("complicances"[All Fields] OR "complicate"[All Fields] OR "complicated"[All Fields] OR "complicates"[All Fields] OR "complicating"[All Fields] OR "complication"[All Fields] OR "complication s"[All Fields] OR "complications"[MeSH Subheading] OR "complications"[All Fields])) OR (("unusual"[All Fields] OR "unusually"[All Fields]) AND ("complicances"[All Fields] OR "complicate"[All Fields] OR "complicated"[All Fields] OR "complicates"[All Fields] OR "complicating"[All Fields] OR "complication"[All Fields] OR "complication s"[All Fields] OR "complications"[MeSH Subheading] OR "complications"[All Fields]).

Eligibility Criteria

Articles that described rare and unusual complications (other than trismus, hematoma, facial nerve paralysis, and ocular complications) following the administration of IAN block documented in English were included. Unusual complications following the administration of local anesthesia using the Gow-Gates technique, the Vazirani-Akinosi technique, and techniques other than IAN block were excluded. All studies published until September 2024 fulfilling the inclusion criteria were included.

Population, Intervention, Comparison, and Outcome (PICO) Framework

The PICO framework was used in this study. For the population, we included patients of any age group who developed unusual complications during the administration of the IAN block. The intervention used was the IAN block. There was no comparison group. The outcome is the resolution of complications.

Selection Process

For the initial selection of studies, the title and abstract of the case reports and case series were reviewed based on the inclusion and exclusion criteria. Then, the full texts of case reports and case series were viewed by all authors.

Data Collection Process

A standardized data extraction form was prepared in Microsoft Excel (Microsoft Corp., Redmond, WA); 3-4 entries were made in Excel and reviewed by an expert. Any disagreement between the authors was resolved by discussion. The following data were extracted: demographics of the study population (age and gender), tooth requiring treatment, medical history, local anesthesia, aspiration, and details of unusual complications.

Results

**Figure 1:**
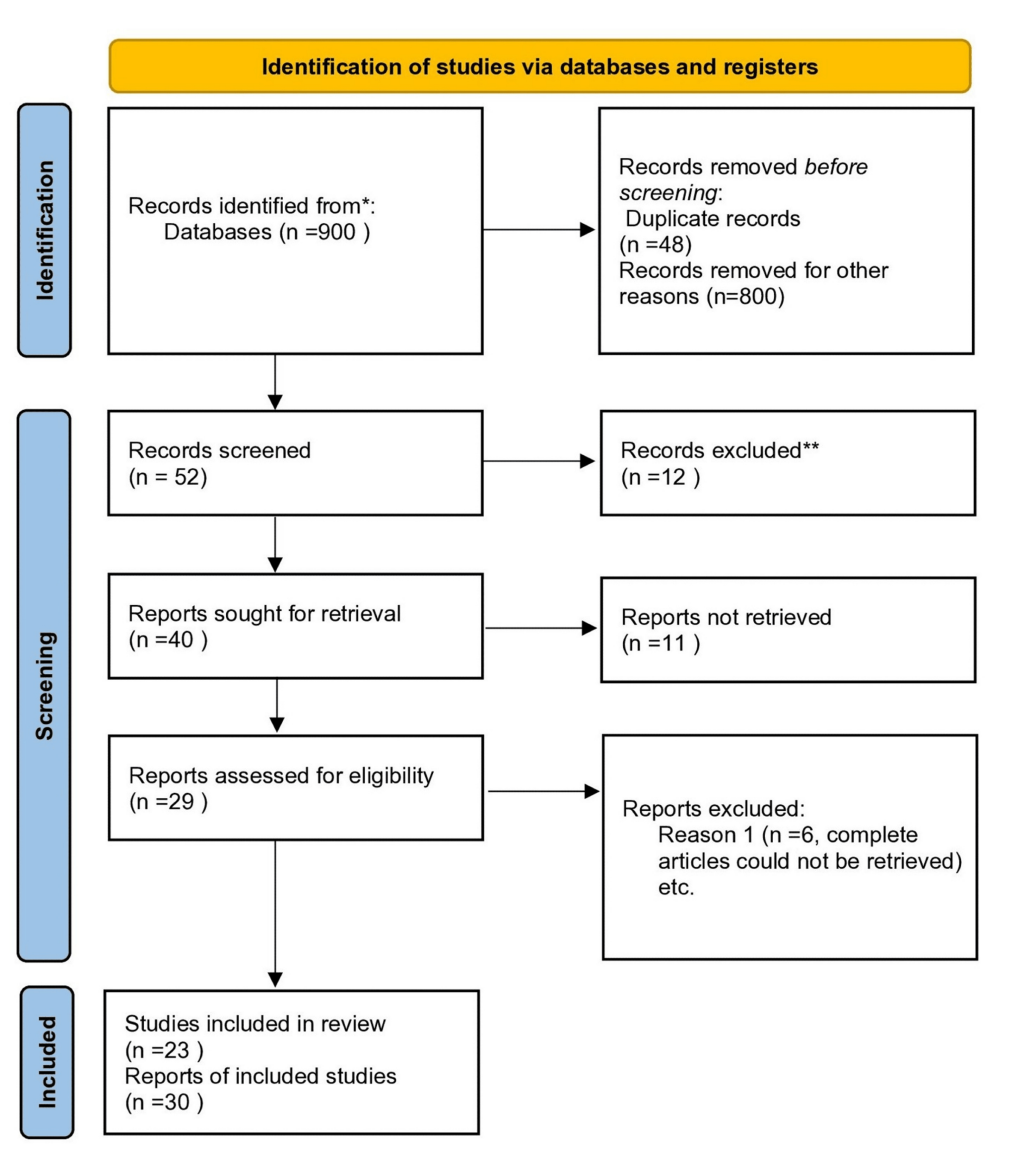
PRISMA 2020 flow diagram Reference: [17] PRISMA: Preferred Reporting Items for Systematic Reviews and Meta-Analyses

A comprehensive search for related articles was done. Duplicates were removed. Titles and abstracts were assessed for the initial selection of articles. Based on inclusion and exclusion criteria, a total of 29 articles were selected, of which six could not be retrieved (Figure 1) [5]. The 23 articles were analyzed (Table 1).

**Table 1:** Details of included cases NA: not available, LA: local anesthesia, IAN: inferior alveolar nerve, TMJ: temporomandibular joint, GP: general practitioner, ASA: American Society of Anesthesiologists

Author and year	Age (years)/gender/mandibular tooth involved for treatment	Medical history	LA aspiration	Time of onset of complications following administration of IAN block	Clinical features and treatment of complications that occurred following the administration of IAN block	Time of recovery of complications that occurred following the administration of IAN block	Dental treatment at the same or next appointment
Campbell et al. (1979) [18]	34/female/third molar	No specific medical history	5 cc of LA; aspiration: NA	Within 2-3 minutes of LA administration	Cervical sympathetic block (Horner's syndrome); treatment of complications: monitoring	2 hours	At a later date, under general anesthesia
Shenkman et al. (1996) [19]	34/male/right first molar	Congenital heart disease	1.8 mL of lignocaine 2% with epinephrine 1:100000; aspiration: negative	20 minutes after the injection (the dental treatment was uneventful)	Permanent neurological deficit, which includes hemisensory syndrome, facial nerve palsy, hearing impairment, and ataxia; treatment of complications: hospital admission	Neurological findings present even at one and a half years	NA
Maini and Preece (2000) [20]	43/male/left lower jaw	NA	NA; aspiration: NA	3 days post-treatment	Herpes zoster infection of the mandibular branch of the trigeminal nerve, lower motor neuron facial palsy, and acute vertigo, herpes zoster oticus, left sensorineural hearing loss; treatment of complications: hospital admission	Recovered	NA
	53/male/right mandibular block	NA	NA; aspiration: NA	10-21 days post-treatment of dental treatment and nasal surgery	Right lower motor neuron facial palsy, herpes zoster oticus, acute vertigo, right sensorineural hearing loss; treatment of complications: hospital admission	Satisfactory recovery of facial palsy at 10 weeks of follow-up; hearing loss continued	NA
Webber et al. (2001) [21]	33/female/left first molar	NA	2% lidocaine and epinephrine 1:100,000; aspiration: NA	Within 1 minute of LA administration	Dizziness, lightheadedness, blanching in the left infra-orbital region, extending to the left side of the nose, lower eyelid, and lip; numbness in the entire left side of the head and face; slight blurring of vision; treatment of complications: monitoring	45 minutes after injection	Treatment done at the same appointment
Sanchis and Peñarrocha (2002) [22]	49/male/NA	NA	1.8 mL of 2% lidocaine with adrenaline 1/100,000; aspiration: NA	A few minutes of LA administration	Swallowing difficulties, foreign body sensation in the throat; velum palatinum paralysis with uvula deviation toward the non-paralyzed side; uvular paralysis; treatment of complications: monitoring	Clinical signs and symptoms disappeared after the anesthetic effect wore off	Treatment done at the same appointment
Uckan et al. (2006) [23]	25/male/first molar	No specific medical history	4% articaine hydrochloride and 1:100.000 epinephrine hydrochloride with 50 mm, 27-gauge needle; aspiration: NA	Seconds after LA administration	Ischemia in the cheek and middle of the forehead, burning sensation in the eye; treatment of complications: monitoring	Approximately 15-20 minutes	NA
	30/female/second molar	No specific medical history	4% articaine hydrochloride and 1:100.000 epinephrine hydrochloride with 50 mm, 27-gauge needle; aspiration: NA	NA	Uniocular blindness, pain in the eye, blanching of the cheek; treatment of complications: monitoring	Approximately 45 minutes	NA
Scott et al. (2007) [24]	28/female/permanent left third molar	No specific medical history	4 mL of 2% lidocaine with 1:80000 epinephrine; aspiration: negative	A few seconds after LA administration	Transient left lateral rectus nerve palsy; skin blanching in discrete area of the upper left lip; treatment of complications: monitoring	Improved markedly prior to the patient leaving the department later that day	Treatment done at the same appointment
Ngeow and Chai (2009) [25]	30/female/left wisdom tooth	No specific medical history	4.4 mL of 2% lignocaine with 1:80,000 adrenaline; aspiration: NA	Just before leaving the clinic, the patient was informed about the symptoms	Numbness in the left TMJ, left auricle, part of the left temple on the ipsilateral side; treatment of complications: monitoring	About an hour	Post-treatment
Paul et al. (2009) [26]	44/male/right third molar	No specific medical history	2% lidocaine with 1:80,000 adrenaline, a 27-gauge long needle; aspiration: negative	Immediately on LA administration	Profound anesthesia and pallor in the right infra-orbital area; pallor in the hard and soft palate area; treatment of complications: monitoring	30 minutes	Treatment done at the same appointment
Bendgude et al. (2011) [27]	4/male/right second primary molar	NA	NA; aspiration: NA	Post-treatment on the same day	Self-inflicted injury; large ulcerative lesion on the lower lip on the right side; large scratch injury on the right side of the chin; treatment of complications: palliative treatment	Two weeks	Post-treatment
Cılasun et al. (2012) [28]	33/male/right second molar	No specific medical history	4% articaine hydrochloride and 1:200.000 epinephrine hydrochloride with 50 mm, 27-gauge needle; aspiration: NA	A few seconds on LA administration	Hoarseness, dysphagia, respiratory difficulty; treatment of complications: monitoring	2 hours	NA
	42/male/ right first molar	No specific medical history	4% articaine hydrochloride and 1:200.000 epinephrine hydrochloride with 50 mm, 27-gauge needle; aspiration: NA	Immediately on LA administration	Hoarseness; treatment of complications: monitoring	Approximately 3 hours	NA
Ezirganli and Kazancioglu (2013) [29]	29/female/third molars	NA	24-mm-long, 27-gauge needle, articaine hydrochloride (40 mg/mL), 1-1.5 mL to IANB and 0.5 mL to lingual nerve; aspiration: negative	Immediately on LA administration	Anemic area on the face covered the lateral nasal wall, inferior orbital ridge, and one cheek; treatment of complications: monitoring	20-30 minutes	NA
	32/female/third molars	NA	24-mm-long, 3-mL syringe, and 27-gauge needle, articaine hydrochloride (40 mg/mL) and epinephrine (adrenaline, 0.012 mg/mL), 1-1.5 mL to the IANB and 0.5 mL to the lingual nerve; aspiration: negative	Immediately on LA administration	Anemic area also covered half of her superior lip; treatment of complications: monitoring	20-30 minutes	NA
Pattni (2013) [30]	22/female/left molar	No specific medical history	2.2 mL of 2% lignocaine with 1:80,000 adrenaline, 27-gauge 35 needle; aspiration: not performed	3 hours post-treatment, oral symptoms developed; approximately 10 hours following administration of the LA, neurological symptoms developed	Burning sensation in her mouth and an area of her chin appeared burnt and blistered; superficial skin erosion in the cutaneous distribution of the left inferior alveolar artery; approximately 10 hours following administration of the LA, the patient developed systemic tremors and numbness, especially of her peripheries and inability to perform simple motor functions; presentation was consistent with systemic complications from the local anesthetic; treatment of complications: the patient presented to her GP	24 hours: systemic and oral symptoms began to subside; 72 hours: systemic symptoms had resolved entirely; 1 month: lesion had healed entirely	NA
Huang et al. (2013) [31]	32/female/left second molar	No specific medical history	27-gauge needle, 30 mm, 1.7 mL 2% lidocaine containing 1:80,000 epinephrine; aspiration: negative	Seconds after LA administration	Horner and Harlequin syndrome; treatment of complications: monitoring	15-20 minutes	Treatment done at the same appointment
Alsukhni et al. (2016) [32]	15/female/left side	No specific medical history	1.5 mL of 2% lidocaine (total dose: 30 mg) and epinephrine 1:100,000; aspiration: no prior aspiration done	By the end LA administration	Several intermittent tonic-clonic convulsions followed by deep coma; treatment of complications: hospital admission	Discharged after a week of admission on complete recovery	NA
Aravena et al. (2016) [33]	21/female/second premolar and first molar	ASA I	27-gauge 0.4 mm × 25 mm needle, 1.8 mL of articaine hydrochloride 4% with epinephrine 1:100,000; aspiration: positive aspiration	At the moment of LA administration	Burning sensation and itching, blanching, pain, and ischemia on the ipsilateral side of the face and oral cavity; treatment of complications: monitoring	10 minutes	Treatment done at the same appointment
Kang and Won (2017) [34]	25/female/left third molar	No specific medical history	1.8 cm^3^ of lidocaine with epinephrine 1:80,000; aspiration: no prior aspiration done	Immediately on LA administration	Facial blanching in the left side of the face (outside of the nose, upper lip, central facial region, and left zygomatic area); pain and discomfort from the middle left facial region to the left orbital region, including the eye; treatment of complications: monitoring	40 minutes	Treatment done at the same appointment
Kumaresan et al. (2017) [35]	57/female/left posterior region	Known diabetic and on oral hypoglycemic medication	1.8 mL of 2% lidocaine hydrochloride with epinephrine 1:80,000; aspiration: negative	Approximately 30 seconds of LA administration	Blanching in the infra-orbital cutaneous region and palatal (hard and soft palate) mucosa and alveolar ridge on the ipsilateral side; treatment of complications: monitoring	20 minutes	Treatment done at the same appointment
Robb (2018) [36]	60/female/left second molar	Traumatic brain injury on the ipsilateral side	Lidocaine; aspiration: negative	Approximately 1 minute after LA administration	Unilateral headache on the ipsilateral side; treatment of complications: hospital admission	Headache lasted for approximately 2 days after the treatment and was resistant to analgesia	Treatment done at the next appointment
Farara and Zakkaria (2020) [37]	11/female/dental procedure	No specific medical history	Lidocaine; aspiration: NA	After LA administration	Status epilepticus, generalized weakness, double hemiparesis; treatment of complications: hospital admission	Recovery within several days	NA
Gillespie and Gunsolly (2020) [38]	59/male/tooth extraction	Hypertension	Lidocaine and epinephrine; aspiration: NA	While receiving LA	Right-sided headache with an episode of palpitations and near syncope; intracranial air embolism; treatment of complications: hospital admission	1-2 days	Dental extraction procedure aborted
Rahpeyma and Khajehahmadi (2020) [39]	25/male/right quadrant	ASA I	Aspiration: NA	NA	Facial blanching and palatal-buccal mucosa whitening occurred; treatment of complications: NA	NA	NA
	22/female/right wisdom tooth	NA	Lidocaine 2% and epinephrine 1:80,000; aspiration: NA	NA	Facial, buccal, and palatal blanching occurred on the side of injection; she felt numbness in the periorbital and lower lip; treatment of complications: NA	10 minutes	NA
Papadopoulou and Anastasakis (2022) [40]	Late 40s/female/routine dental procedure	No specific medical history	Articaine 4% plus epinephrine were initially administered (40 mg/mL plus 1:100000, respectively, one ampoule of 1.7 mL) with a needle 0.30 × 25 mm, 30-gauge; after 5-10 minutes, because of inadequately achieved anesthesia in the tongue, a further administration of mepivacaine 3% (30 mg/mL one ampoule of 1.8 mL) was infused in the same spot as the first one; aspiration: NA	30 minutes after administering LA	Difficulty in swallowing, nasal speech, uvula deviated to the left side; numbness around the external auditory meatus; transient glossopharyngeal nerve palsy; treatment of complications: monitoring	3 hours and gradually subsided	Post-treatment
Das et al. (2023) [41]	52/male/left side second molar	NA	NA; aspiration: NA	NA	Pain, fullness, and numbness in the left ear; bleeding in the external auditory meatus; treatment of complications: monitoring	2 hours	Dental treatment terminated for the day
Hassan et al. (2023) [42]	26/female/left first molar	Mild gastritis	1.8 mL of LA containing lidocaine 2% (1:100,000 adrenaline); aspiration: NA	10 minutes after LA administration	Numbness, altered sensation, inability to move the entire left upper extremity; area and muscles innervated by the median and ulnar nerves were affected; treatment of complications: shifted to the emergency department	8 hours	NA

The patients presented with various conditions such as Horner's syndrome, herpes zoster infection, blanching of the face, self-inflicted injury, hoarseness, convulsions, headache, intracranial air embolism, glossopharyngeal nerve palsy, and bleeding in the external auditory meatus. The most common local anesthetics used were lignocaine and articaine. Treatment ranged from monitoring in mild cases to hospital admission in severe cases.

Risk of Bias Assessment

The Joanna Briggs Institute (JBI) Critical Appraisal Checklist for Case Reports was used to assess the case reports [43]. The case reports are of moderate to high quality. Although most of the information was reported in the cases, some information related to either the patient's medical history, technique (aspiration and needle size), or whether the treatment was done at the same appointment was missing. Information related to medical history and technique can help in assessing if there is any correlation between these factors and complications reported, so necessary precautions can be taken to prevent these complications.

Discussion

The IAN block is a commonly performed dental nerve block to anesthetize the lower teeth and soft tissues in the hemimandible region. Anatomical variations and technical failures can lead to complications when IAN block is performed. Although rare, unusual complications have been noted during the administration of IAN block. These complications were noted in patients irrespective of age, gender, and specific medical history. In a few cases, pre-existing medical conditions/previous history of trauma and anxiety were predisposing factors for complications that occurred during IAN block administration [36,42]. Awareness of the rarely occurring complications can help dentists in diagnosing, reassuring patients, and planning further treatment. Although most of the complications are benign in nature, they can make the patient anxious. Monitoring, referral to higher centers, and hospital admissions are the various treatment protocols followed in the management of these rare complications.

Various theories have been proposed in relation to the complications of administering IAN block. These include drug overdose leading to systemic toxicity, inadvertent intravascular injection (either intra-arterial or intravenous), or injection in the vicinity of the central nervous system (CNS), leading to neurological complications [19,31-33,36-38].

Vasoconstriction of blood vessels, such as the maxillary artery, results in skin and mucosa blanching. It occurs due to the action of epinephrine on α-adrenergic receptors in the periphery of the skin and mucosa. Sympathetic vasoconstrictor fiber stimulation can lead to the contraction of blood vessels, resulting in facial ischemia [21,23,24,26,29,30,33-35,39].

Accidental injection of local anesthesia directly into the carotid sheath or subperineural spread of local anesthesia results in cervical sympathetic block or stroke-like symptoms [18,42]. The effect of local anesthesia at the ipsilateral brachial plexus via retromandibular vein, external jugular vein, and plexus of veins around the vertebral artery leads to symptoms that are similar to a stroke, where patients complain of ipsilateral upper extremity paralysis and paresthesia [42].

A high IAN block can lead to the spread of local anesthetics toward the glossopharyngeal nerve, leading to uvular paralysis. When the block is injected at a higher level, the mandibular nerve and supply to the tensor veli palatini can be affected, leading to uvular paralysis [10]. Diffusion of local anesthesia close to the glossopharyngeal nerve in the carotid triangle through the parapharyngeal space can lead to this complication [40].

Numbness in the auricle could be the result of auriculotemporal nerve anesthesia during the administration of IAN block, which rarely happens. Anatomical variations such as the origin of the auriculotemporal nerve at a lower level, connecting branch with IAN, and spread of local anesthesia through the masticatory space to reach the superior location are the various causes mentioned [25,29]. Laceration and ear bleeding result from the superior movement of the needle in the parotid gland vicinity during the administration of IAN block [41].

A self-inflicted injury in the area of the lip was noted. These kinds of injuries are usually noted in children. These injuries are usually self-resolving and heal with palliative care, unless they get infected secondarily [27].

Individual anatomical variation of the sympathetic nerve may allow the anesthetic solution to be delivered to ectopic sites, leading to temporary complications. These complications subside once the local anesthesia wears off [28].

Activation of a latent virus can lead to herpes zoster infection during the administration of IAN block. Stress and dental surgical treatment can lead to reactivation of these viruses. Treatment involves the administration of antivirals and steroids [20].

It is advocated that aspiration must be performed during the administration of IAN block. Intravascular injection can lead to failure of anesthesia. Administration of local anesthesia in blood vessels has been associated with undesirable systemic effects. Hemodynamic effects, especially in patients with medical conditions such as cardiovascular diseases, must be considered. A greater concentration of vasoconstrictor and rapid deposition of local anesthesia increase these possibilities. Studies have shown that the percentage of positive aspiration varies, which can be as high as 20% [44]. In some of the cases above, complications occurred even when aspiration was negative. Even when the initial aspiration is negative, slight tilting of the needle due to patient movement during the final administration of IAN block could lead to intravascular injection.

Lidocaine and articaine are the most commonly used local anesthetics in the above cases. Lidocaine has been a commonly used local anesthetic in dentistry and has been found to be effective and safe. In recent times, local anesthetics, such as articaine, have gained popularity. Because of its effectiveness and safety levels, it is used for routine dental procedures [45].

Precautions must be taken by dentists while administering IAN block to avoid unusual complications. Medical history must be considered, and stress must be managed during dental procedures. The dentist must have thorough knowledge of anatomy, and the correct technique and guidelines must be considered during the administration of the block. Aspiration and slow administration of the solution are recommended. These measures lessen the amount of solution that reaches the systemic circulation, hence minimizing the risk of complications [34].

Patients should always be informed about the unusual complications that can occur due to the administration of IAN block. In case an event occurs, the dentist must be knowledgeable of the differential diagnosis and must effectively manage patients. The dentist must reassure the patient.

All dental procedures must be terminated in case any complication occurs following the administration of IAN block. The condition must be diagnosed, and the patient must be monitored. If the patient is calm and the condition is stable, dental treatment can be performed after obtaining patient consent. In some cases, additional treatment, such as hospital admission, must be initiated depending on the complication. If the complaint does not subside, postponing the treatment can be considered [36].

Limitations

This systematic review includes only case reports and case series.

## Conclusions

Although rare, unusual complications can occur following the administration of the IAN block. Patients should always be informed about the same before the start of the same treatment. The dentist must be knowledgeable about the complications. Precautions must be taken to avoid them. In case complications occur, the condition must be diagnosed immediately. Patients must be informed about the same and reassured so that they remain calm. Although most complications resolve and dental treatment can be completed at the same appointment with patient consent, referral to medical centers may be necessary in certain cases. Rare complications must always be considered when administering an inferior alveolar nerve block.
